# Diversity and Content Reliability Among Dermatologist Influencers on TikTok: A Cross-Sectional Study

**DOI:** 10.7759/cureus.106063

**Published:** 2026-03-29

**Authors:** Madison S Meyer, Shoshanah Lasry, Khalid Zakaria

**Affiliations:** 1 Family Medicine, Wayne State University School of Medicine, Detroit, USA; 2 Medicine, Nova Southeastern University Dr. Kiran C. Patel College of Osteopathic Medicine, Davie, USA; 3 Internal Medicine, Henry Ford Health System, Novi, USA

**Keywords:** diversity in dermatology, lgbtqia+, sexual and gender minority physicians, skin of color, social media

## Abstract

TikTok has surged in popularity as a primary source of entertainment for Americans, with many physicians leveraging the platform to disseminate up-to-date medical information. Within this landscape, dermatology has emerged as one of the most sought-after medical subjects, leading to the prominence of numerous board-certified dermatologists as influential figures. However, despite this visibility, dermatology remains among the least diverse medical specialties.

This cross-sectional study investigates patterns of following and content reliability on TikTok, particularly concerning dermatological information, based on the racial, gender, and sexual orientation diversity of top influencers. Through qualitative and quantitative analysis of the top 55 dermatologist influencers on TikTok, based on data collected on October 10, 2022, we assess the demographics and video characteristics, with a focus on follower count.

Our study found that content produced by Latinx and African American dermatologists demonstrated relatively higher DISCERN scores, although overall content reliability across all groups was low. Additionally, our findings underscore a significant lack of minority representation among dermatology influencers on TikTok, particularly among Latinx, African American, and LGBTQIA+ physicians.

This lack of diversity may limit the availability of culturally representative dermatologic information, highlighting a potential gap that has been associated in prior literature with inequities in care and health outcomes. By addressing these diversity gaps, we can work towards fostering more inclusive and equitable healthcare environments on social media platforms.

## Introduction

Dermatology persists as one of the least diverse medical specialties, a fact underscored by statistics revealing the stark underrepresentation of racial, sexual, and gender minorities within the field [1]. African Americans constitute a mere 3% of board-certified dermatologists, despite comprising 12.8% of the American population [2]. Similarly, Latinx individuals make up only 4.2% of dermatologists, compared to their 16.3% representation in the general populace [3].

These disparities extend beyond mere demographics, manifesting in tangible health outcomes. Members of minority populations, such as those identifying as African Americans and Latinx, experience increased mortality rates and poorer prognoses for dermatological conditions, like melanoma, despite having lower incidence rates [4]. Moreover, patient-physician racial concordance has been shown to correlate with improved healthcare outcomes, including increased patient participation and satisfaction [5].

The field of dermatology also grapples with a lack of diversity among sexual and gender minorities (SGM). Currently, SGM individuals remain vastly underrepresented among dermatologists [6]. This lack of diversity has significant implications, as SGM individuals often face discrimination within healthcare settings, contributing to suboptimal care and health outcomes [7].

The rise of social media, particularly platforms like TikTok, has further highlighted these disparities. TikTok has emerged as a primary source of medical information, with dermatology content garnering significant attention [8]. However, it remains unclear whether the most highly followed dermatologist influencers on TikTok reflect the same patterns of underrepresentation of Latinx, African American, and lesbian, gay, bisexual, transgender, queer/questioning, intersex, and asexual (LGBTQIA+) physicians, observed among board-certified dermatologists. A lack of representation among dermatologists can perpetuate healthcare disparities while reducing minority access to culturally appropriate dermatologic knowledge [7].

Thus, this study aims to investigate patterns of following and content reliability among top dermatology influencers on TikTok, assess the demographics and video characteristics of these influencers, and inform strategies for promoting diversity, equity, and inclusion in dermatology and social media. By shedding light on these disparities, we seek to inform strategies for promoting diversity, equity, and inclusion within dermatology and social media influencer communities.

## Materials and methods

We conducted a cross-sectional, mixed-methods study to assess race, gender, sexual identity, and content type among the 55 largest board-certified dermatologist TikTok influencers. Influencer ranking was based on follower count at a single time point. Follower counts were recorded at the time of data collection and may fluctuate over time. For this study, we assessed all eligible accounts included in the top 55 at the time of data collection.

Qualitative and quantitative data were collected on October 10, 2022. Data extraction was performed using the TikTok search engine. The keyword “dermatology” was searched, and board-certified dermatologists were reviewed in descending order based on follower count at a single time point. Board-certified dermatologists were included if they met the inclusion criteria and were in the top 55 at the time of data collection. Accounts were excluded if the owner was not a licensed physician or if they fell outside the top 55 at the time of data collection.

Gender, race, and sexual identity were collected from information publicly available on each dermatologist’s TikTok profile or websites linked in their TikTok bio. Demographic data were based on publicly available, self-disclosed information, which may have resulted in incomplete demographic classification. If the dermatologist did not publicly self-identify their demographics on their TikTok profile or linked websites, their demographic was marked as “not publicly disclosed.” Attempts were not made to contact dermatologists for demographic information.

The three most recent videos from each account were reviewed. Videos were then analyzed and placed into one of three categories: “Educational,” “Personal,” or “Advertisement.” Videos deemed “Educational” included any video discussing dermatologic conditions, treatments, procedures, or related topics. “Personal” videos included videos discussing matters that did not fall into the “Educational” category. Videos that were created to advertise dermatologic products or services were placed in the “Advertisement” category.

Educational videos were evaluated using the DISCERN instrument, adapted for short-form video content. Applicable DISCERN criteria were assessed for each video, and each video was assigned a DISCERN score on a scale of 1 to 5, with higher scores indicating greater reliability [9,10]. Two independent reviewers evaluated all videos, and discrepancies were resolved through consensus discussion.

Lastly, the number of views, likes, comments, and total account followers was recorded for each dermatologist. Differences were analyzed between underrepresented in medicine (URM) and non-underrepresented in medicine (non-URM) dermatologists. URM was defined as individuals who publicly self-identified as African American or Latinx. Differences were also analyzed between female and male dermatologists. Descriptive statistics were performed. Two-tailed, independent-samples t-tests were used to compare URM versus non-URM dermatologists and male versus female dermatologists. Statistical testing was not performed for Latinx dermatologists due to insufficient sample size (n = 1), and findings are reported descriptively. p-values < 0.05 were considered statistically significant. All analyses were performed using Google Sheets (Google LLC, Mountain View, CA, USA; version 2022).

This study used publicly available data and did not involve human subjects, as defined by the U.S. Department of Health and Human Services; therefore, institutional review board approval was not required.

## Results

When reviewing the demographics of the top 55 dermatologist TikTok influencers, we found that one (1.8%) publicly self-identified as Latinx, and 10 (18.2%) publicly self-identified as African American. Overall, 11 (20.0%) of dermatologist influencers publicly self-identified as URM (Table 1). Within the top 55 dermatologist influencers, we did not identify any Latinx or African American males (Table 1). Of note, 29 (52.7%) of the identified influencers were Caucasian, while 15 (27.2%) identified as Asian (Table 1).

**Table 1:** Race of dermatologist TikTok influencers (N = 55)

	Total (N = 55), n (%)
Latinx	1 (1.8)
African American	10 (18.2)
Caucasian	29 (52.7)
Asian	15 (27.2)

Male dermatologists demonstrated the highest mean views per account (2,276,724.90), while Asian dermatologists had the second-highest mean views per account (1,776,950.40) (Table 2). URM dermatologists demonstrated higher mean DISCERN scores compared to non-URM dermatologists. African American dermatologists demonstrated significantly higher mean DISCERN scores compared to non-URM dermatologists (mean 2.0 vs. 1.6, p = 0.0016). Comparisons involving Latinx dermatologists were not subjected to statistical testing due to insufficient sample size (n = 1) and are therefore presented descriptively (Table 2). Female dermatologists demonstrated higher mean DISCERN scores compared to male dermatologists (1.8 vs. 1.6); however, this difference did not reach statistical significance (p > 0.05).

**Table 2:** Characteristics of the top 55 dermatologists on TikTok

	Mean Views (n)	Mean Likes (n)	Mean Comments (n)	Mean Bookmarks (n)	Mean DISCERN (n)	Mean Total Likes (n)	Mean Followers (n)
Latinx	917,894.0	50,065.0	448.0	907.0	2.0	158,800.0	6,696.0
African American	44,404.0	364.0	5.9	46.2	1.9	686,057.4	54,241.6
Caucasian	1,468,714.6	2,821,975.9	1,375.2	11,624.4	1.6	24,867,258.6	1,046,298.5
Asian	1,776,950.4	119,845.5	1,808.7	4,803.4	1.6	25,410,356.2	1,403,393.5
Female	1,055,204.8	2,183,992.9	1,123.2	8,988.1	1.8	8,596,862.0	635,148.0
Male	2,276,724.9	212,537.1	2,320.6	5,104.7	1.6	39,014,900.0	1,591,782.3

Out of all videos included in the analysis, 109 out of 165 (66.0%) were educational videos, and 18 of 165 (10.9%) were personal videos (Table 3). The remainder of the videos analyzed were advertisements.

**Table 3:** Category of the three most recent videos posted by the top 55 dermatologists on TikTok as of October 10, 2022

	Educational, n (%)	Treatment Advertising, n (%)	Personal, n (%)
Latinx	3 (100)	0 (0)	0 (0)
African American	15 (50)	12 (40)	3 (10)
Caucasian	62 (71)	18 (21)	7 (8)
Asian	29 (64)	8 (18)	8 (18)

Of the top dermatologists (N = 55), 37 (67.2%) were female, and 18 (32.8%) were male (Table 4). Female dermatologists had lower means for every category compared to males, except for DISCERN score, where females had a higher mean score (1.8 vs. 1.6) (Table 2). Female dermatologists had an average of 635,148 followers, while male dermatologists had an average of 1,591,782.30 followers (Table 2).

**Table 4:** Gender of dermatologist TikTok influencers (N = 55)

	Male, n (%)	Female, n (%)	Other Gender Identity, n (%)
Latinx	0 (0)	1 (100)	0 (0)
African American	0 (0)	10 (100)	0 (0)
Caucasian	15 (51.7)	14 (48.3)	0 (0)
Asian	3 (20)	12 (80)	0 (0)

None of the dermatologists within the top 55 publicly self-identified as LGBTQIA+ or as a gender identity other than male or female at the time of data collection (Table 4).

## Discussion

The dermatology specialty faces a growing diversity, equity, and inclusion challenge when it comes to providing quality care for underserved minorities [11]. As patients increasingly turn to TikTok for healthcare information, increasing the number of URM physicians within the dermatology specialty may allow for more content regarding different presentations of dermatologic conditions among different races, as most posts recorded had educational intent. These findings should be interpreted within the context of a single time-point analysis conducted in October 2022, which may not reflect current influencer dynamics on TikTok. Additionally, the application of the DISCERN instrument to short-form video content presents inherent challenges, as the tool was originally developed for written health information.

None of the top board-certified dermatologist influencers on TikTok publicly identified as Latinx male or African American male. The only Latinx physician in the top 55 dermatologists ranked near the bottom in terms of number of followers. The respective rankings were number 52 and 55 out of the top 55 dermatologists. Our findings are similar to other studies demonstrating an overall lack of URMs in dermatology [1]. Out of the top 10 most-followed TikTok dermatologists, there were no URMs. The highest-ranked URM dermatologist ranked number 20 out of the top 55 dermatologists on TikTok. 

Minimal exposure to posts regarding dermatologic diseases that present in skin of color (SOC) may contribute to unequal distribution of knowledge regarding dermatologic diseases that manifest in people of color [12,13]. This limited exposure and understanding of dermatological disease manifestations may contribute to differences in awareness of dermatological disease manifestations and timing of care-seeking [14]. Improving representation in dermatology may begin at the medical school and residency level, which can help increase the number of URM physicians accepted into dermatology residency programs. Dermatology residents can continue this trend by becoming influencers on TikTok and creating content that spreads awareness of how dermatologic conditions can differ in SOC.

Although only 20% of dermatologists on TikTok were URMs, content produced by URM dermatologists demonstrated modestly higher DISCERN scores compared to non-URM dermatologists, though overall reliability remained low across all groups. While descriptive analysis suggested higher mean DISCERN scores among African American and Latinx dermatologists, interpretation of these findings, particularly for Latinx dermatologists, is limited by small sample sizes (n = 1) and should be interpreted with caution. African American dermatologists demonstrated higher mean DISCERN scores, which reached statistical significance (p < 0.05).

None of the top dermatologists on TikTok publicly identified as LGBTQIA+. Our results align with other studies that demonstrate low numbers of dermatologists from diverse backgrounds [1,15]. This lack of exposure to LGBTQIA+-specific dermatologic content may contribute to existing knowledge gaps [16]. Many LGBTQIA+ patients face barriers when attempting to seek dermatological care due to previous occurrences of discrimination when being treated by providers [17]. Additionally, a lack of exposure to these topics may cause future physicians to be biased when treating LGBTQIA+ patients [17]. Increasing visibility of LGBTQIA+ individuals in dermatology can help improve the care of LGBTQIA+ patients by creating and disseminating content that addresses these knowledge gaps.

Limitations 

A major limitation of this study is that data were collected at a single time point on October 10, 2022. Given the rapidly evolving nature of TikTok, including changes in influencer rankings, engagement, and content trends, these findings may not reflect current patterns and should be interpreted as a cross-sectional snapshot rather than a representation of ongoing dynamics. Additionally, another limitation of this study is that not every dermatologist on TikTok was accessible during the search for the word “dermatology,” which may have contributed to different representations in the ranking of the top influencers. TikTok search results are influenced by algorithmic personalization, which may limit reproducibility and introduce selection bias. Demographic data were based on publicly available, self-disclosed information or inference, which may have resulted in misclassification or incomplete representation, particularly for individuals who did not publicly disclose demographic information. Since there was only one Latinx dermatologist out of the top 55 dermatologist influencers on TikTok, the mean calculation of the data for Latinx physicians may have been skewed. Given the small number of publicly self-identified Latinx dermatologists (n = 1), DISCERN comparisons for this subgroup were descriptive only and not intended for statistical inference. The adaptation of the DISCERN instrument for short-form video content may not fully capture content quality, as the tool was originally developed for written materials. An additional limitation is that fluctuating TikTok trends may cause changes in the ranking of dermatologists based on new followers and users unfollowing accounts. These limitations highlight the need for future studies using longitudinal or updated datasets to better assess evolving trends in dermatology content on social media platforms.

## Conclusions

This study builds on past work regarding the persistent lack of inclusion of URM physicians in the dermatology community on TikTok. Our findings also bring to light issues with limited physician diversity on social media platforms and how this highlights a potential gap in the reliability of dermatologic education being disseminated on these platforms. Without increased representation of URM dermatologists among highly visible TikTok influencers, educational content addressing SOC and atypical presentations may remain limited. Better representation in dermatology starts at the medical school and residency level to inspire URM physicians to enter into dermatology and to be equitably selected into dermatology residencies. From there, increasing the number of URM physicians in the field may help increase physicians of color on platforms like TikTok and allow for the creation of more inclusive and reliable content. Similarly, the methods of this study can be applied to other medical specialties to assess physician diversity and representation, as well as the reliability of posted content on social media.

Future work can assess factors that may play into differences in the reliability of content among dermatologist influencers. There is also a need for further research to understand barriers that may limit URM and SGM physicians from becoming as visible and popular on social media. Improvements to diversity in dermatology and efforts to uplift minority physician voices on social media may help create more equitable health education for marginalized patient populations.
